# Individual differences in experienced and observational decision-making illuminate interactions between reinforcement learning and declarative memory

**DOI:** 10.1038/s41598-021-85322-2

**Published:** 2021-03-15

**Authors:** Batel Yifrah, Ayelet Ramaty, Genela Morris, Avi Mendelsohn

**Affiliations:** 1 Sagol Department of Neurobiology, University of Haifa, 3498838 Haifa, Israel; 2 The Institute of Information Processing and Decision Making (IIPDM), University of Haifa, Haifa, Israel; 3 Rappaport Faculty of Medicine and Research Institute, Technion - Israel Institute of Technology, 31096 Haifa, Israel

**Keywords:** Reward, Human behaviour

## Abstract

Decision making can be shaped both by trial-and-error experiences and by memory of unique contextual information. Moreover, these types of information can be acquired either by means of active experience or by observing others behave in similar situations. The interactions between reinforcement learning parameters that inform decision updating and memory formation of declarative information in experienced and observational learning settings are, however, unknown. In the current study, participants took part in a probabilistic decision-making task involving situations that either yielded similar outcomes to those of an observed player or opposed them. By fitting alternative reinforcement learning models to each subject, we discerned participants who learned similarly from experience and observation from those who assigned different weights to learning signals from these two sources. Participants who assigned different weights to their own experience versus those of others displayed enhanced memory performance as well as subjective memory strength for episodes involving significant reward prospects. Conversely, memory performance of participants who did not prioritize their own experience over others did not seem to be influenced by reinforcement learning parameters. These findings demonstrate that interactions between implicit and explicit learning systems depend on the means by which individuals weigh relevant information conveyed via experience and observation.

## Introduction

Decision making relies on the integration of multiple sources of information gathered both from experiential behavior and by observation of others’ behaviors, which are learned both explicitly and implicitly. Consider for example trying to decide what to order in a restaurant—one could either wait to see what others choose, or rely on pleasant and unpleasant memories of previous orders, as well as on personal preferences developed over time to reach a decision. Thus, preferences are shaped by implicit and explicit action-outcome associations acquired by observation and/or experience, and therefore by an interplay between implicit trial-and-error learning and declarative representations of relevant information. Indeed, learning and memory systems in the brain interact to enhance adaptive behavior. A prominent example of this is the interaction between reinforcement learning signals and declarative memory formation, whereby implicit learning may either facilitate or compete with declarative memory formation^1,2^. The current study explored how sources of information, which differ in their relevance to optimal learning may affect implicit and explicit learning systems, as well as the interaction between them.

Overall, choice mechanisms rely on a comparison between the predicted values of all available options. Several valuation systems have been proposed, which come into action depending on the type of behavior to be carried out, the task at hand, and the internal state of the organism^3^. The values of actions may be learned through trial and error by means of reinforcement learning^4^, which relies on reward prediction errors. These signals, which are mediated by midbrain dopaminergic neurons^5,6^, play a role in the updating and representation of predicted reward values in the brain throughout the course of learning. Alternatively, the options could be evaluated by a goal-directed valuation search process, in which the long-term consequences of each of the options are explicitly recalled and compared for value^7^. Studies have implicated the ventromedial prefrontal cortex (including adjacent medial orbitofrontal cortex) in encoding expected value representations^8–11^. Note, however, that computing these requires explicit knowledge of the consequences of each option, which, in turn, may be stored in the form of semantic memory, but may also be the result of episodic memories of single specific events^12–14^.

Restricting learning to the consequences of one’s own actions may, in many cases, be insufficient and even maladaptive. Consequently, many species, including humans, often learn from others by means of observation^15,16^. Observing actions and outcomes of others can be incorporated into one’s own knowledge by updating values according to their experiences, to inform decision-making^17^. Both experiential and observational learning have been posited to rely on similar computational mechanisms^18^. However, fMRI studies have shown that experiential and observational reward learning engage distinct brain regions^16,19,20^, consistent with findings from Parkinson’s disease patients, who appear to be able to learn by observation that does not involve cue-feedback associations^21^. There are cases, however, where observing the behaviors of others may be irrelevant or even detrimental to one’s goals. Such instances should require the observer to either ignore the action-outcome associations learned from observing others, or to differentiate between experienced and observational information when updating values that affect decisions.

The formation of long-term memories is often biased towards prioritizing the encoding of high- over low-value information^22,23^. Indeed, the perceived relevance of information has been shown to enhance allocation of attentional resources critical to successful memory formation^24^. As such, declarative items are more likely to be remembered when associated with high value outcomes^25^. While the benefits of mnemonic processes in choice behavior and planning have been recently explored^12,26^, it is unclear whether similar prioritization occurs during the encoding phase of experiences that convey information from multiple sources. Our goal in this study was to explore whether the value judgments of unique experiential and observational episodes affect implicit and explicit measurements of learning and memory. Specifically, we examined how incidental memory formation is affected by observational versus active reinforcement learning. To this end, we designed a novel reward learning task that included both active (experienced) and observed trials and the presentation of event-unique pictures, followed by a recognition memory test of the pictures. By applying a reinforcement learning model on a subject-by-subject basis, we were able to dissociate participants who either learn similarly from action and observation, or separate the learning signals from these two sources. We demonstrate that interactions between implicit and explicit learning systems depend on the type of learning strategies adopted by the learner. In particular, subjects who assigned different weights to their own experience and to that of others showed enhanced specific memory gains for episodes involving significant reward prospects. On the other hand, subjects who did not prioritize their own active experience over observed trials did not exhibit enhanced memory of potentially rewarding episodes.

## Results

The current study set out to explore how the learning of cue-outcome associations, updated through direct experience and observation, affects incidental memory of declarative information presented throughout. We therefore designed a decision-making task that included interleaved active (experienced) and observed trials, and presented trial-unique pictures on each trial. Memory for the pictures was tested by a surprise recognition memory test after completion of the experimental session.

### Two types of learners

**Figure 1:**
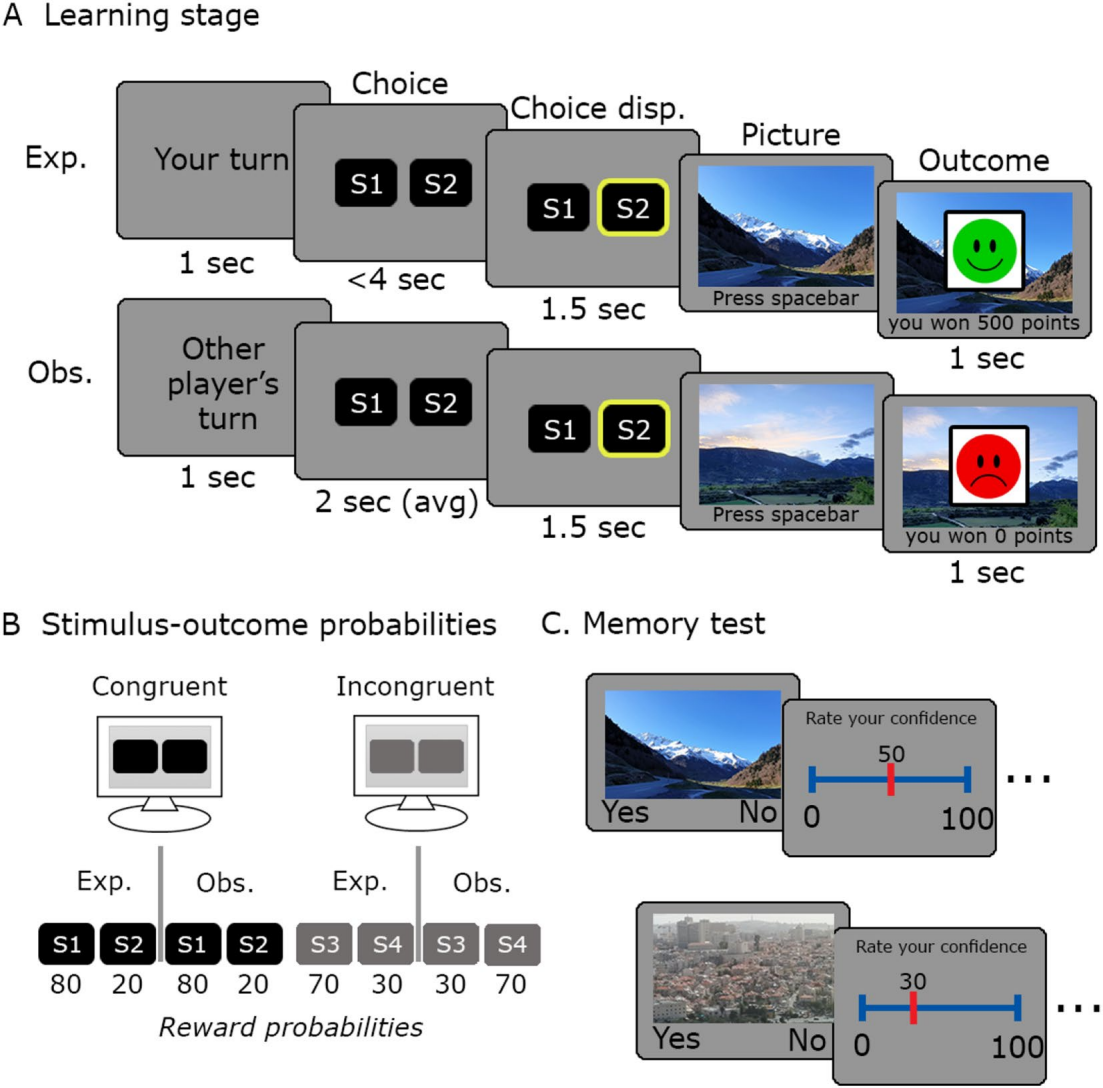
Experimental design. (**A**). The decision-making task included ‘experienced’ trials, in which participants actively chose between two visual cues, each associated with a predefined reward probability; and ‘observed’ trials, in which participants observed the choices of what they were told was a second player (and was actually a computerized simulation). Before revealing a reward or neutral outcome for each choice, a trial-unique picture, unrelated to the reward learning task, was presented in center of screen. (**B**). Two types of stimulus pairs were used in the task: in the ‘Congruent pair’, the cue-outcome contingencies were similar for experienced and observed trials (80:20 reward contingency), and in the ‘Incongruent pair’ trials, cue-outcome contingencies were reversed (70:30 vs. 30:70 reward contingency). (**C**). A surprise recognition memory test was administered immediately after the decision-making task. For each image presented on screen, participants were required to determine whether it appeared during the decision-making part of the task or not, and to indicate their confidence in their decision. *S* stimulus, *Exp.* experienced, *Obs.* observed, *disp.* display.

The task consisted of two trial types (Fig. 1A): experienced trials, in which participants actively chose between one of two visually presented cues, each associated with a predefined reward probability; and observed trials, in which the participants observed the choices of a second player (actually a computerized simulation). We used two pairs of cues (Fig. 1B). In the congruent pair, the reward contingencies of the cues (80:20) were identical in experienced and observed trials. In the incongruent pair, the contingencies (70:30) were reversed between the two players. This resulted in an easy, unambiguous pair, and a more difficult and ambiguous pair.

**Figure 2:**
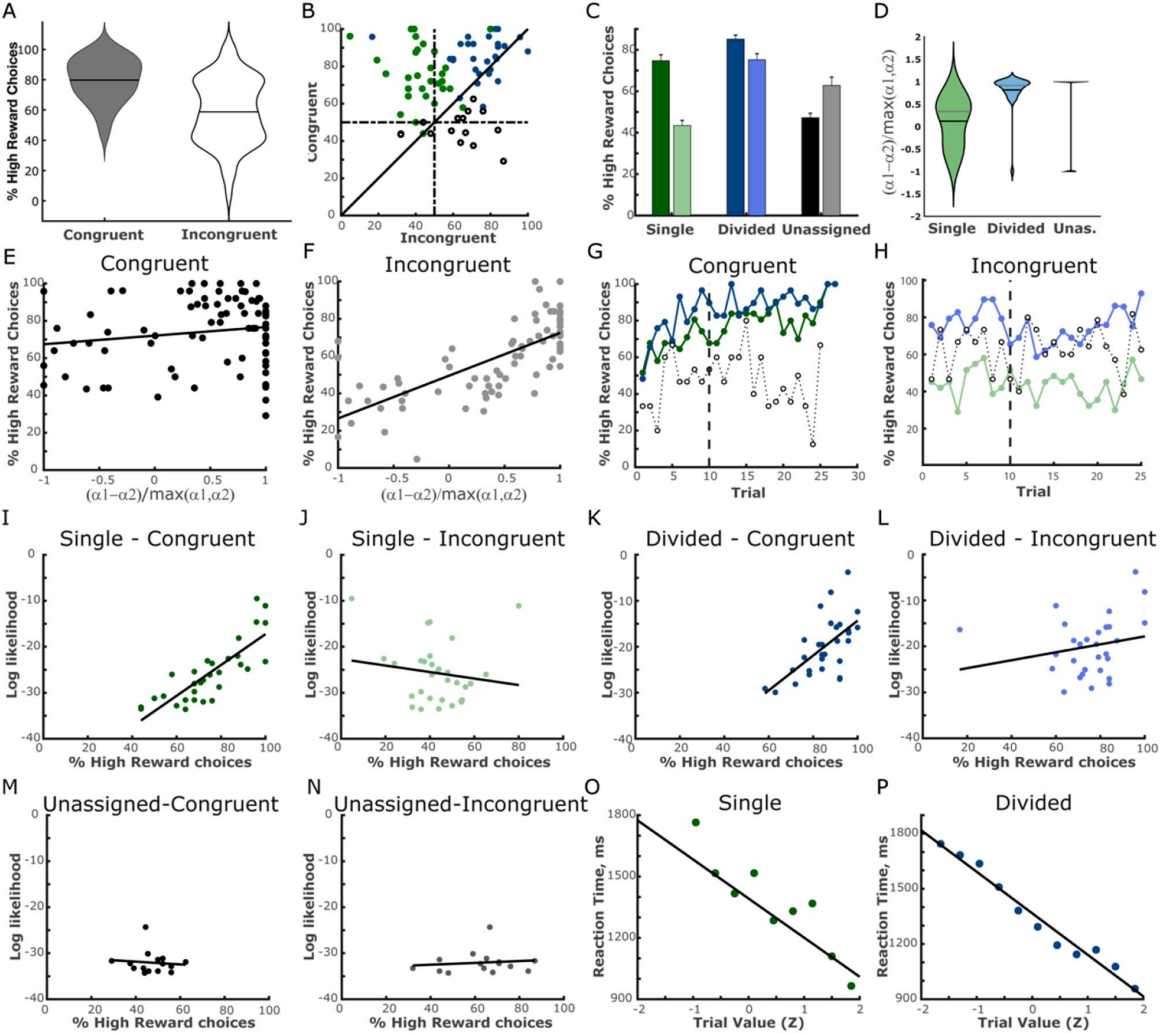
Individual differences in learning strategies. (**A**) Distributions of the proportion of high-reward choices in congruent (dark grey) and incongruent (white) conditions, showing a bimodal distribution of high-reward choices in the incongruent pair condition. The mean of each distribution is indicated by a black line. (**B**) Individual scores on congruent and incongruent trials of subjects assigned to the ‘single strategy’ (green), divided strategy (blue) and unassigned (empty marks). (**C**) Proportion of high-reward choices divided according to learning strategy, depicting single strategy model scores for congruent (dark green) and incongruent (light green) pairs, scores of the ‘divided strategy’ group for congruent (dark blue) and incongruent (light blue) conditions, and unassigned participants (in dark and light grey). The lowest high-reward choice scores were evident in the ‘single strategy’ group for the incongruent pair. (**D**) Distributions of relative differences between learning rates calculated of experienced and observed conditions for each group. (**E**–**F**) Correlations between relative learning-rate differences (using a two learning-rate model) and learning scores for congruent (left) and incongruent (right) pairs. (**G**–**H**) Learning curves of the single (green), divided (blue) and unassigned (grey) groups for congruent and incongruent stimuli pairs. The dotted line at trial 10 represents the end of the predominantly experienced stage. Panels (**I**–**N**) depict correlations between subjects’ proportion of high-reward choices and the quality of the model fit (log likelihood of subjects’ choices) for each group and each condition. Note that log-likelihoods in panels (**I**) and (**J**), as well as in (**K**–**L**) and (**M**–**N**) are identical, as the model fit was performed across congruence conditions. (**O**–**P**) Mean reaction-times of stimulus choices during the decision-making task binned as a function of calculated high-reward stimulus values for the single-strategy group (r = − 0.91) and the divided-strategy group (r = − 0.98).

Figure 2A depicts the subjects’ performance in congruent and incongruent conditions, as indicated by the proportion of trials in which the subjects chose the cue associated with the higher reward probability. As the congruent condition was easier to learn both in terms of the cue-reward contingencies and congruence among players, performance was expectedly superior in these trials relative to the incongruent ones (t (74) = 4.49, *p* < 0.0001). This is corroborated by reaction times, where participants were overall slower to respond on incongruent trials (RT = 1150.8 ± 37.9 ms and 1254.9 ± 42.4 for the congruent and incongruent conditions, respectively, t (74) = 3.66 *p* < 0.0001). In the congruent trials, subjects’ choices of the high-value rewards ranged between 40 and 100% of the trials, with a large concentration between 80 and 100% (Fig. 2A, left). In contrast, scores on the incongruent trials followed a bimodal distribution (Fig. 2A, right). Thus, the participants’ behavior on incongruent trials appeared to comprise two distinct sub-types: participants who incorporated the observed trials into their estimation of the values of the cues (effectively lowering their scores in the incongruent trials), and those who ignored the observed trials, resulting in more high-probability cue choices in the incongruent trials.

To capture this difference between subject types, we modeled each subject's choice sequence according to four alternative reward learning (RL) models (see “Methods” section). Briefly, the single learning-rate model (‘single’) applies a single learning rate to all trials (experienced and observed) of both pairs, the experienced/observed model (‘divided’) differentiates between experienced and observed trials and applies independent learning rates to each. The congruent/incongruent model (‘pairwise’) assumes independent learning rates to each pair regardless of experienced/observed conditions, and an additional model allowing for four separate learning rates for the experienced/observed, and congruent/incongruent conditions. To choose the approach that best describes the population, we compared the four models by their cumulative Bayesian Information Criterion^27^ (BIC), which showed that the divided model outperformed all other models (cumulative BIC scores for single, divided, pairwise, and four learning-rate models: 4690.7, 4542.7, 4784.7, and 4946.5, respectively). Although our design did not have sufficient power to accurately capture the four-rate model (see confusion matrix in Fig. S1), and therefore cannot rule out the possibility that the subjects actually differentiate between all trial types, these results indicate that a critical factor in the subjects’ behavior is the capacity to assign different learning rates to experienced and observed trials. Therefore, to account for the observed individual differences between our subjects, we used the individual BIC scores to assign each subject to one of three groups: the ‘single strategy’ group, comprising subjects who applied the same learning rate to their own (experienced) and their partners’ (observed) trials; the ‘divided strategy’ participants, who applied different learning rates to experienced and observed trials; or ‘unassigned’, if the likelihood of the chosen model was not higher than chance. The parameters estimated for each subject are presented in Supplementary Table S1, along with the log-likelihood and BIC scores. Overall, 60/75 subjects were successfully modeled by one of the two first strategies. The proportions of subjects best described by each of the two chosen models were approximately similar: the ‘single strategy’ group included 31 participants, whereas the ‘divided strategy’ group included 29 participants. The remaining 15 participants were not assigned for neither of the strategy groups, as their model fit scores were not different than chance (see Table S3).

Figure 2B depicts individual subjects’ choice scores on congruent and incongruent trials, separately for each of the strategy groups. The division by strategies captures differences in the subjects’ performance: while members of the ‘divided strategy’ group performed well on trials of the congruent and the incongruent pairs, those of the ‘single strategy’ group were inferior in the incongruent pair. We applied a mixed-model analysis to the participants’ choices to compare the performance of the single and divided strategy groups by pair type (congruent/incongruent). We found that the ‘divided strategy’ group outperformed the ‘single strategy’ group across conditions (F (1,58) = 67.11, *p* < 0.0001, Fig. 2C). Moreover, learning was superior for the congruent pair compared to the incongruent pair across the groups (F (1,58) = 62.33, *p* < 0.0001). Crucially, the benefit of congruence was higher for the ‘single strategy’ group, as evidenced by an interaction between pair type and strategy (F (1,58) = 16.68, *p* < 0.0001). Similar effects were obtained upon adding the unassigned participants (main effect for pair (F (1,72) = 12.48, *p* < 0.001); main effect for group (F (2,72) = 48.7, *p* < 0.0001); pair x group interaction effect F (2,72) = 2.904, *p* < 0.0001)).

From Fig. 2B, C it is also apparent that most of the subjects of the ‘unassigned’ group displayed superior performance in incongruent trials compared to congruent. This behavioral pattern may seem odd, unless these participants treated both the congruent and incongruent stimulus pairs as if they were incongruent (such that they adopted a choosing strategy opposite to the ‘second player’). It is important to note here that although subjects were told explicitly that all profits would be divided equally between them and the ‘second player’, they were not told anything else about the second player’s game. Some players may have assumed that they were playing the same game, while others may have assumed that they may be playing different games. This prompted us to examine whether participants’ behavior of the entire sample may be explained by models that take into account not only separate learning-rates for experienced versus observed conditions, but also their hypothetical interpretation of the observed stimulus-feedback contingencies. We therefore examined four different models (see also Table S2): The first model (I) treats both pairs as though they were congruent, i.e., as though the reward contingencies are identical for the subject and the ‘second player’. This model is effectively the same as the original two-learning-rate model, with different learning rates for experienced and observed trials. The next two models differentiate between pairs. Model II assumes that the incongruent pair is indeed incongruent, and therefore that the reward contingencies for this pair are reversed for the subject and the second player. Model III reverses contingencies on observed trials only of the congruent pair. In the fourth model, the contingencies in the observed trials are reversed for both pairs. High fit scores for this model would indicate that participants treated both pairs as incongruent.

This approach nicely accounted for a large fraction of the participants we dubbed ‘unassigned’, as many of these subjects were best fitted by model IV (7/15), which treats both pairs as incongruent. It is noteworthy, however, that this modeling approach yielded overall inferior model-fit scores compared to the original one versus two learning-rate approach (see Table S1 for parameters and Figure S4 for analysis of behavioral measures based on this separation).

To examine whether the ‘divided strategy’ group simply benefited from applying two different learning rates, or whether they indeed updated selectively experienced trials, and observed trials to a lesser degree, we calculated the relative differences in learning-rates associated with experienced and observed trials $\frac{{\alpha_{1} - \alpha_{2} }}{{\max \left( {\alpha_{1} ,\alpha_{2} } \right)}}$. While the ‘divided strategy’ group had very large differences in learning rates, those of the ‘single strategy’ group were much lower, showing similar learning rates for experienced and observed trials (Fig. 2D). When considering the entire sample (except the unassigned group, who did not sufficiently fit the RL models), we found that the relative learning-rate difference corresponded with a tendency to choose the high-reward stimulus in incongruent trials (r = 0.78, *p* < 0.0001, Fig. 2E). Since choice scores were relatively high for the congruent pair across the entire sample, no such correlation was found in these trials (r = 0.16, N.S., Fig. 2F). An alternative separation between subject groups, based on relative learning rate difference, rather than individual BIC scores is presented in Figure S5.

In addition to learning scores across the entire task, we examined the group percentage of high-reward stimulus choices on a trial-by-trial basis for each group separately (see Fig. 2G, H). Whereas both the single and divided strategy groups showed a gradual increase in correct choices for congruent stimuli, only the divided group acquired the contingencies of the incongruent pair. Note that the first 20 trials of the task (i.e., the first 10 trials for each pair) consisted almost entirely of experienced trials, such that the actual incongruence between the participants and their counter ‘second players’ effectively began after several trials (as depicted in Fig. 2G, H by dashed vertical lines). In fact, for the incongruent stimulus pair, a dip in performance after trial 10 is apparent in the divided group, after a series of overall successful choices (possibly by chance). While the single strategy group remained around chance level throughout (as would be expected by averaging their own experienced contingencies with the opposite observed ones), performance increased in individuals assigned to the divided-strategy group, presumably since they opted to dismiss the choice-feedback information displayed in the observed trials, and remain loyal only to their own experience. Posterior predictive checks using the posterior distributions of the parameters estimated by the models indeed captured these behaviors^28^ (see Fig. S2).

To gauge the ability of the models to capture the participants’ RL performance, we computed correlations between the model fit of the best model for each subject and the subjects' choice scores (i.e., percentage of choices of the cue with highest reward outcome, see above), separately for each pair. As expected, significant correlations were found in the congruent condition in both the ‘single strategy’ group (r = 0.816, *p* < 0.0001) and the ‘divided strategy’ group (r = 0.619, *p* < 0.001, Fig. 2I, K). In the incongruent pair, only subjects in the ‘divided strategy’ group, whose RL strategy was beneficial in these trials, exhibited a significant correlation between the subject score and the model fit (r = 0.419, *p* < 0.05, Fig. 2J, L). Note that there was an outlier participant from the divided-strategy group in the incongruent condition, which was not included in the correlation calculation. Conversely, the ‘single strategy’ group, employing a strategy that was detrimental to learning in incongruent trials, showed no correlation between the model fit and scores on the incongruent pair (r = − 0.149, N.S., Fig. 2J). As to the unassigned group, the model-fit scores, which were low by definition, did not correlate with RL scores in neither of the pairs (Fig. 2M, N). We therefore conclude that the model fit of each participant is indicative of the subjects’ RL engagement.

It is noteworthy that despite the difference in model assignment for the single- and divided-strategy groups, basic task-related behavioral measurements were comparable across these groups. As such, reaction times (RT) of stimulus choices did not differ between the groups (single group mean of median RTs = 1252.3msec ± 58.3, divided group = 1160.7 ± 56.6, t (58) = 1.12, N.S.). The choice RT for the unassigned group was also not different than that of the other groups (1129.5 ± 97.5, One-Way ANOVA, F (2,72) = 0.92, N.S.).

Further validation supporting the use of the learning model to the behavioral outcome of the task at hand comes from the observation that the estimated values of rewarding stimuli predicted reaction-times to choices made in corresponding (experienced) trials (Fig. 2O, P). Specifically, the higher the estimated values, the faster the choices were made (R = − 0.91 and R = − 0.98 in the single and divided groups, respectively). This encouraged us to further examine the relationship between trial-by-trial estimated values and memory performance, as described below.

### Memory performance is comparable on average across all groups and conditions

**Figure 3:**
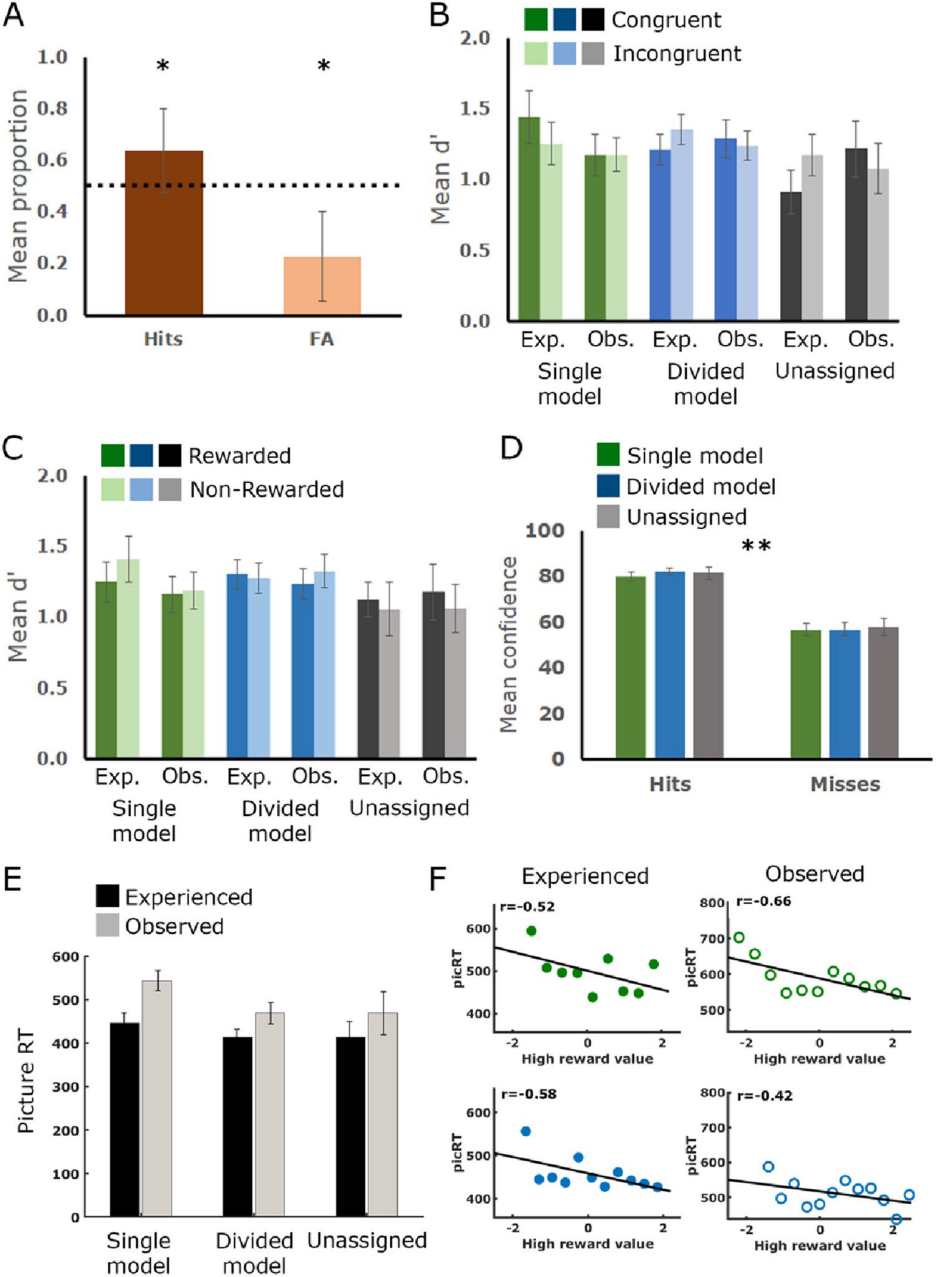
Memory performance by strategy groups, pairs, and trial outcome. (**A**) Memory performance averaged across participants and conditions was significantly higher than chance. (**B**) Memory strength (*d*′) was comparable on average across groups, conditions, and pairs. (**C**) Memory strength (*d*′) was comparable for rewarded and non-rewarded trials across groups and conditions. (**D**) Confidence ratings were significantly higher for hits than misses across the three groups. (**E**) Mean reaction times for pressing the spacebar key to reveal the trial outcome upon picture presentation in each trial. Across all three groups, RTs during experienced trials were shorter than those during observed trials. (**F**) The picture-RTs correlated with the estimated values of the high-reward stimuli for both single (green) and divided (blue) strategy groups, such that the higher the value associated with a given trial, the faster the response.

A surprise memory recognition test of the pictures presented during the gambling task was administered immediately after completion of the decision-making task (Fig. 1C). The proportions of hits were greater than chance (mean ± S.E. = 0.64 ± 0.14, t (74) = 8.5, *p* < 0.0001), and false alarm rates fell well below chance (FA: 0.23 ± 0.15, t (74) = − 15.74, *p* < 0.0001), with a significant difference between hit rate and false alarm rate, indicating overall intact memory (t (74) = 19.049, *p* < 0.0001, Fig. 3A). No difference in memory performance (measured as d-prime (*d*′), i.e. the normalized difference between hit-rate and false-alarm rate for each participant, see “Methods” section) was found between pairs (F (1,72) = 0.281, N.S.), between experienced versus observed trials (F (1,72) = 0.004, N.S.), nor between strategy groups (F (2,72) = 0.327, N.S., Fig. 3B), as examined by a mixed model ANOVA test. Similarly, no differences in memory strength (d′) were found between rewarded and non-rewarded trials (Fig. 3C), neither for group type across conditions (F (2,72) = 0.405, N.S.) nor for experienced versus observed trials across groups (F (1,72) = 1.68, N.S.). Confidence ratings for hit responses were much higher than subjective memory strength for misses (F (1,72) = 240.8, *p* < 0.0001), yet were comparable across groups (F (2,72) = 0.108, N.S.) (Fig. 3D).

The time frame in which the picture was presented occurred between the subjects’ choice and their key-press to present the feedback (see also Fig. 1). We therefore tested whether this time differed systematically between groups and between conditions (Fig. 3E). This examination showed that all three groups responded faster in experienced versus observed trials (F_1,72_ = 45.3, *p* < 0.0001), but the groups did not differ from one another (main effect for group—F_2,72_ = 2.46, N.S.). Since the subjects’ response revealed the feedback regarding the choice made at that trial, we examined whether this response time was related to trial value. Figure 3F depicts feedback response time as a function of high-reward stimulus value in experienced and observed trials. In both groups and both conditions response time was negatively correlated with trial value (single group, experienced, r = − 0.52, *p* < 0.01, observed, r = − 0.66, *p* = 0.06; divided group, r = − 0.58, *p* < 0.05 and r = − 0.42, N.S, for experienced and observed trials, respectively), indicating faster responses (and therefore shorter picture study times) on trials which the subjects considered more profitable.

### Trial values correlate with subsequent memory of pictures presented in concurrent trials

RL model fit was previously shown to be negatively correlated with declarative memory performance^29^. To examine whether the learning strategies revealed in our subject pool similarly interfered with declarative memory formation, we computed correlations between memory strength (*d*′) and RL model fit of the best model found for each subject across participants for each group separately. The ‘divided strategy’ group demonstrated a non-significant but marginal negative correlation between model fit and subsequent memory performance (r = − 0.33, *p* = 0.071), while no correlation was observed in the ‘single strategy’ group (r = 0.017, N.S., see scatter plots in Fig. S3). The two correlation coefficients did not, however, differ significantly (Fisher Z = − 1.32, *p* = 0.093).

**Figure 4:**
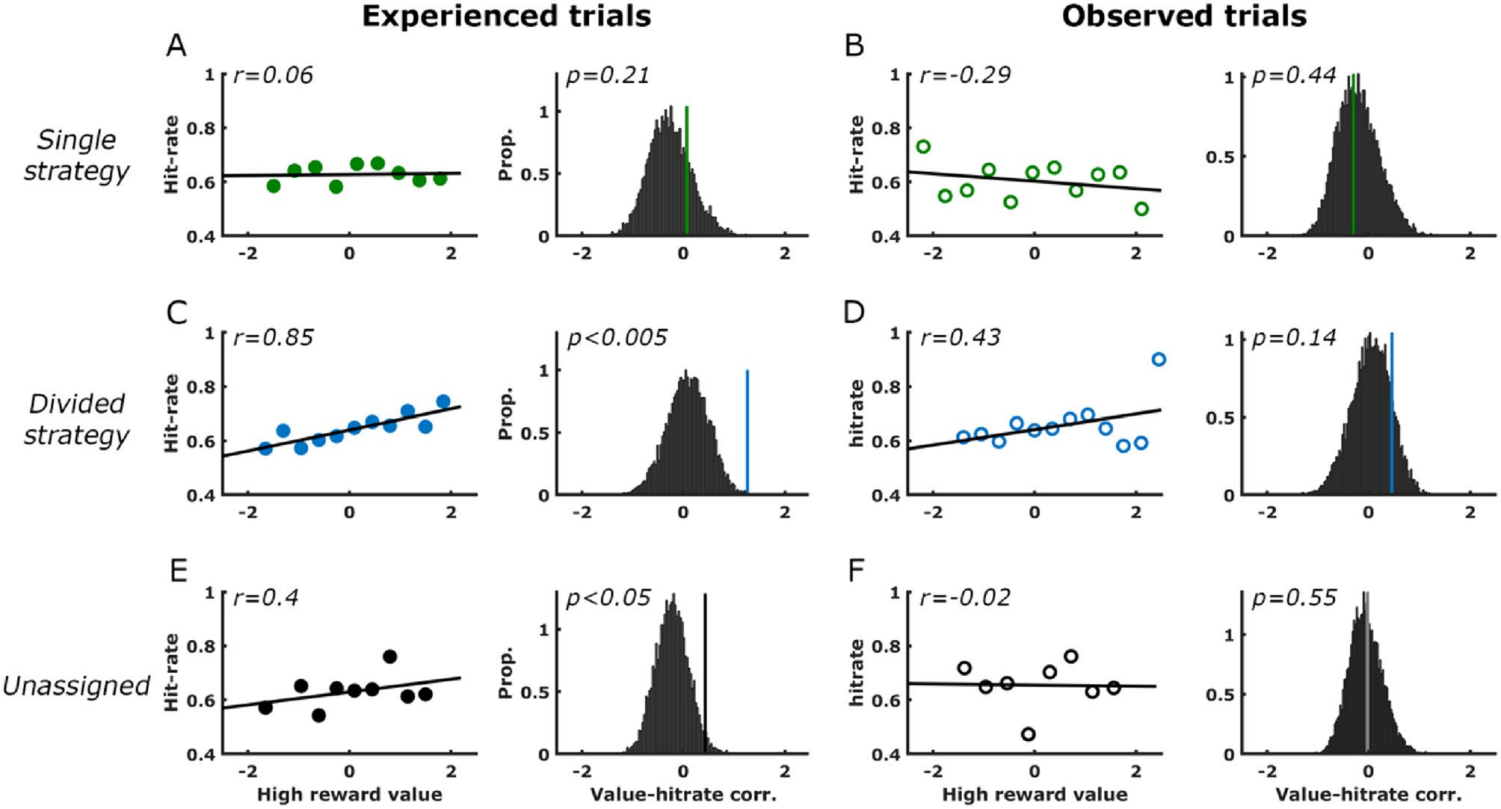
Memory performance is positively associated with maximum cue-values during experienced trials of the ‘divided strategy’ group. Scatter plots here and below depict correlations between memory performance (measured as proportion of hits) averaged across participants with the maximum stimulus-value available in a trial. (**A**, **C**, **E**). Correlations between memory performance and high reward values during *experienced* trials for the single-strategy, divided-strategy, and unassigned groups, respectively. All correlations were tested against 10,000 shuffles of each subject’s data (right panels). Actual correlations are represented by vertical lines in relation to the histogram of shuffled data. (**B**, **D**, **F**) Similar analysis as above for *observed* trials.

In attempt to examine the possible relationship between trial-by-trial model values and corresponding subsequent memory performance, we computed the relationship between the stimulus-related values updated for each and every trial, and memory performance for the images presented at each trial. Since the memory test was related to images that appeared immediately following choices, and prior to the feedback regarding the trial’s outcome, we examined the effect of the expected value of the cue most likely to be rewarded, on memory for events on each trial. Our choice of the high-reward value as an indicator of trial value was validated by its correlation with decision reaction time in the experienced trials (see Fig. 2O, P) and with picture-feedback reaction time (Fig. 3F). Figure 4 depicts memory performance as a function of the value of the best option available in each trial, separately for experienced (Fig. 4A, C, E) and observed trials (Fig. 4B, D, F) for the three subject groups. For subjects belonging to the ‘divided strategy’ group, the value of the best option in a trial was predictive of memory for pictures presented immediately after the choice. Importantly, this effect was more prominent in experienced trials, (Fig. 4C) where active choices were made, and not when merely observing choices made by the alleged ‘second player’ (Fig. 4D) (experienced trials: r = 0.85, *p* < 0.005; observed trials: r = 0.43, *p* = 0.14). The correlation coefficients in experienced and observed trials were compared using a Meng's Z test^30^ for dependent correlations, which confirmed that they were different (*p* < 0.05). By contrast, for subjects of the ‘single strategy’ group, no relation was found between the value and memory performance on either trial type (experienced trials: r = − 0.03; observed trials: r = − 0.07; Fig. 4E–F). Direct comparison between the correlation coefficients in the 'single' and 'divided' groups using Fisher Z transformation confirmed that these were significantly different (*p* < 0.05). Other model variables, such as the reward prediction errors (both signed and unsigned), or values of the chosen stimuli did not appear to be related to memory performance.

**Figure 5:**
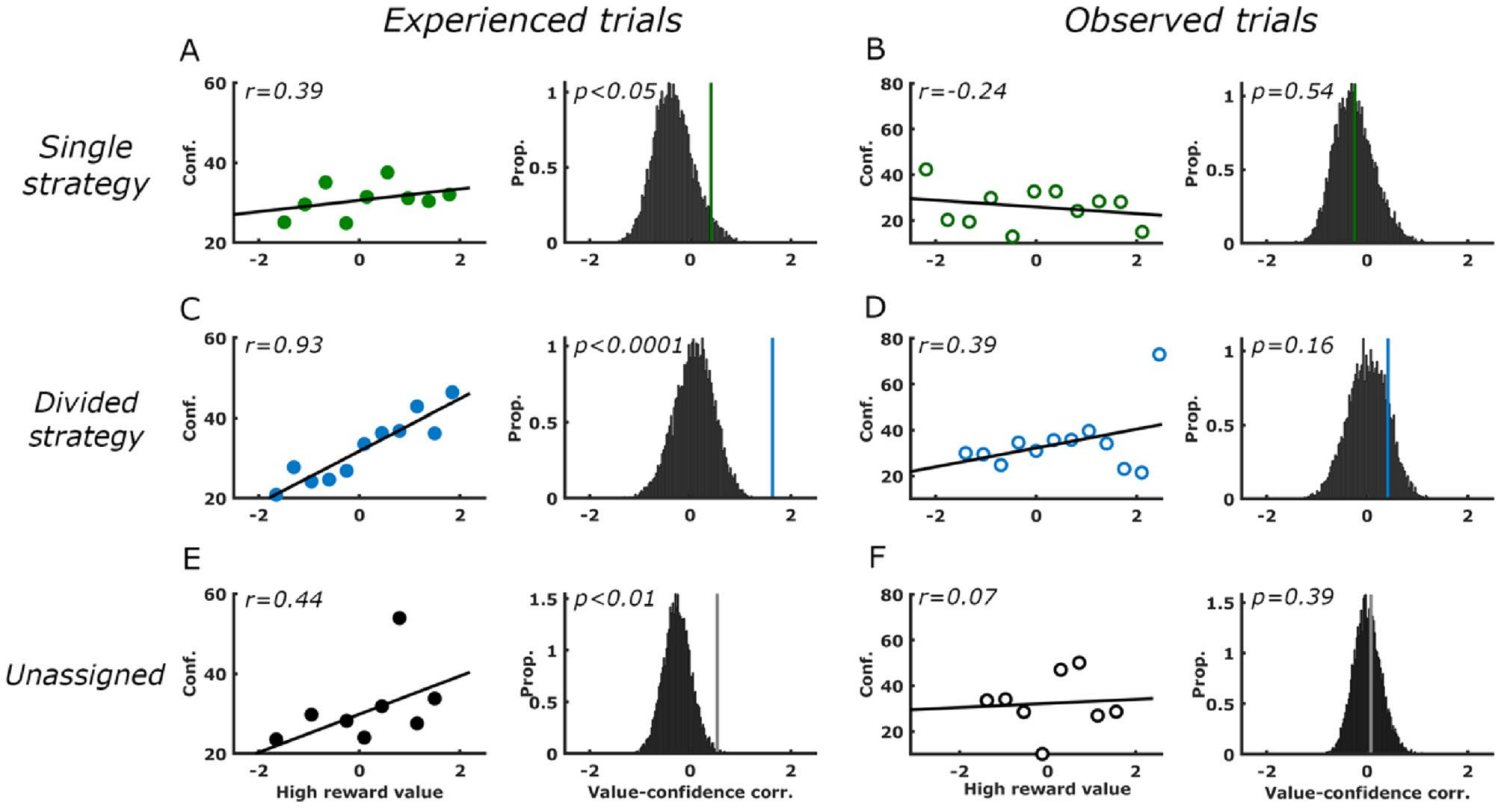
Confidence ratings are positively associated with maximum cue-values during experienced trials. Scatter plots here and below depict correlations between subjective memory strength (measured as mean confidence ratings) with the maximum stimulus-value available in a trial. (**A**, **C**, **E**). Correlations between memory performance and high reward values during *experienced* trials for the single-strategy, divided-strategy, and unassigned groups, respectively. All correlations were tested against 10,000 shuffles of each subject’s data (right panels). Actual correlations are represented by vertical lines in relation to the histogram of shuffled data. (**B**, **D**, **F**) Similar analysis as in left panels for *observed* trials.

To supplement the abovementioned findings regarding the relationship between estimated reward-predictive stimulus values and actual memory performance (i.e. hit-rates), we tested the subjective memory reports (confidence ratings) by trial value. For assessing these relations, confidence scores for each subject were arranged on a scale from − 100 (positively sure of a wrong miss answer) to 100 (positively sure of a correct hit answer). As depicted in Fig. 5, the high-reward stimulus values are strongly predictive of confidence ratings for recognition of corresponding images. This is true for experienced trials (Fig. 5, left panels), but not for observed trials (right panels). Note that positive relationships between stimulus values and confidence ratings are apparent in all three groups, yet only in the divided-strategy group does the significance level survive correction for multiple (six) comparisons.

## Discussion

The current study explored how reward-based learning efficacy, value representation, and source prioritization of learning signal (experienced vs. observed), affect future memory of information presented during the course of trial and error-based decision making. By applying a model-fit approach, we classified participants into three groups—those who distinguished between their own trials (experienced) and the trials of their partners (observed) (thus termed the ‘divided strategy’ group), those who did not distinguish between experienced and observed trials (the ‘single strategy’ group), and a third unassigned group, who did not fit either of the models. We found that although overall memory performance for items presented during the decision-making task was similar, the two main groups (single- and divided- strategy) differed in the interaction between incidental episodic memory and the RL process. In the ‘divided strategy’ group, memory strength was negatively correlated with the RL model-fit, indicating a tradeoff between RL and declarative memory. Conversely, participants who did not separate between learning in the experienced versus observed trials did not show a similar pattern. Moreover, in the divided strategy group, memory for visual items was associated, on a trial-by-trial basis, with the best predicted values on the same trials, but only in experienced events, in which the values were relevant for their decision making. Overall, our results shed light on the interactions between RL performance and incidental declarative memory formation, by demonstrating that the interaction between the underlying systems is affected by individual learning strategies regarding the accumulation of information relevant to adaptive decision making.

### Interactions between reward-based learning and memory formation depend on learning strategy

Our experimental paradigm presented the participants with two types of social learning scenarios: a congruent condition, wherein experienced and observed actions yielded similar outcomes, and an incongruent condition, in which the outcomes of experienced and observed actions yielded conflicting associations. Previous studies have demonstrated that the observation of others’ actions and the consequences of those actions may be updated and incorporated into the observers’ behavioral repertoire^17^. Thus, computational models have demonstrated that value updating can rely on actions of others and ensuing outcomes^18,31^. Such observational learning can take the form of imitation, whereby individuals mimic actions that were frequently made by observed others, as well as emulation, where inferences are made regarding other agents’ goals or intentions^32,33^. Our design could not differentiate between these two forms of observational learning, yet by fitting behavior to models that accounted for distinct learning parameters, we were able to distinguish between participants who incorporated observational information into their own decision making process versus those who did not. This was rendered possible by presenting observational information that in some cases concurred with experience and in other cases disagreed. Thus, participants were best served by learning from others in a selective manner. Indeed, the alternative models we applied to behavior differentiated between participants who incorporated observational information to update cue values from those who were selective in learning from others. Moreover, the strategies that were captured by the participants’ actions yielded different interactions between cue values and declarative memory formation on a trial-by-trial basis.

Participants in both groups exhibited intact learning in the congruent pair condition. The success of both groups to adequately learn the cue-outcome associations in these trials is in line with studies showing that observing choices of others can be used as a source of prediction error computations that can benefit one’s actions^16^. Indeed, brain regions involved in representations of others’ choices and outcomes share some overlap with those that respond to learning from experience^17,20^. By contrast, the two groups diverged sharply in their success on trials of the incongruent pair. The tendency of participants to either rely on observational feedback or to discard it was captured in our model by pitting two RL models against each other—one with a single learning rate for experienced and observed trials^34^, and one with different learning rates for each trial type^16^, separately for each subject. We note that the incongruent pair was, by design, also more difficult to learn due to the higher uncertainty dictated by the contingencies. This property may have led some subjects to the adoption of a single strategy, due to difficulty in tracking the contingency differences between experienced and observed trials, leading to disadvantageous learning from the observed choices, and others to the adoption of a divided strategy, disregarding the observed choices to reduce noise. This feature enabled us to explore the impact of learning strategies on the interactions between RL and incidental memory formation.

### Value representation of relevant cues positively scales with memory formation

Model fitting is often used in order to arbitrate among models to explain behavioral patterns of a sampled population^35^. Here we used model fitting to assign different models to different individuals, based on the number and nature of their assessed learning-rates. The fitting of a reward-based learning model on an individual basis enabled us to estimate trial-by-trial values assigned to each cue. We found that the values of the cues associated with higher reward delivery probability in each trial (as estimated by the model) were predictive of memory for pictures presented following the choice, before feedback presentation. This was despite the fact that picture viewing time was negatively correlated with cue value. The modulation of memory by trial value was apparent only for participants who successfully distinguished between their own choice-outcome associations and their counterparts (‘divided strategy’ group), and only in trials that were relevant for learning (i.e., experienced trails). This effect is reminiscent of observations showing that reward expectation tends to enhance hippocampal-dependent memory^36–38^, possibly via midbrain dopaminergic innervation^39,40^. Furthermore, our findings add to previous studies that point in favor of cooperation between implicit and explicit learning systems, leading to an additive effect on memory^41,42^. For instance, a recent study demonstrated a correspondence between memory for pictures associated with what the authors termed as image reward prediction-errors^41^, akin to maximum cue values in our study. As in that study, the appearance of the pair of cues in our experiment informed the participants regarding the trial’s potential profitability, which affected memory for the event as a whole. Similarly, reward prediction-error strength was shown to correspond to declarative memory of images and words presented during learning^2,42,43^. In our study, the positive relationship between trial values and memory was apparent only in trials that were deemed relevant for successfully updating the predictive value of associated outcomes. As such, participants who failed to discern between experienced and observational learning signals did not show this effect. It is noteworthy that despite this group difference, memory performance was comparable on average across groups, implying overall similar allocation of attention resources and cognitive effort.

As recently suggested, decision making and planning are guided both by explicit and implicit memory systems^12,26^. Here we shed light on the process by which information that may be indicative of profitable decision-making events is prioritized, leading to stronger memories of those events. Thus, those who succeed in discerning between relevant and irrelevant sources of information for increasing future reward are also able to give precedence to contextual information associated with high value events. The prioritization of relevant information is in turn reflected in forming memories of the event as a whole, including unique declarative information that can be later retrieved upon demand. We provide evidence here that indeed memories for information acquired during instances predictive of rewarding outcomes are perceived with stronger confidence, possibly indicating recollection versus mere familiarity^44^.

An alternative explanation for the reported findings is that rather than basing performance on reinforcement learning strategies, the presented cues could have been evaluated directly by a goal-directed valuation search process, in which the long-term consequences of each of the options are explicitly recalled and compared for value^7^. This explicit recall may be biased towards prioritizing the encoding of high- over low-valued information^22,23^. Older adults, for instance, were shown to be highly skilled in the engagement of adaptive prioritization which permits allocating more resources to high value information^23^. Our results suggest significant variability in the way individuals prioritize resource allocation, not only in terms of which information to encode, but also in assigning differential learning weights to implicit or explicit systems as well as information source.

In conclusion, it is increasingly acknowledged that reward-based learning and episodic memory systems interact in various manners^45^. The current study illuminates this issue by demonstrating facilitation of memory formation during high-value decisions on a trial-by-trial level. By applying a RL model-fit approach to each participant, we were able to discern learners on the basis of how they determine relevant from irrelevant information, and demonstrate that details presented by information sources deemed relevant to adaptive behavior have a better chance of being committed to memory.

## Methods

### Participants

Seventy-nine healthy volunteers participated in this study. The study was approved by the local ethics committee of the University of Haifa, and informed consent was obtained prior to the experiments. All methods were carried out in accordance with relevant guidelines and regulations. Data from four participants were excluded due to non-compliance and technical issues in the memory test stage, leaving seventy-five subjects (47 females) between the ages of 19 and 47 (mean age of 24.9 ± 4.8).

### Behavioral procedure

The experiment consisted of two stages—a decision making task that required the learning of cue-outcome associations, followed by a recognition memory test of pictures presented during the task. The task was administered simultaneously to two participants in two different rooms. Immediately prior to task onset, participants were introduced to their alleged partner (the ‘second player’), and were told that they will play together, each performing half of the trials, and receiving half of their combined rewards. In practice, each participant played against a computerized simulation of a participant (see below).

### Decision making task

The task consisted of 100 trials, fifty ‘experienced’ trials and fifty ‘observed’ (see Fig. 1). On experienced trials, participants chose between two visual cues, each associated with a different probability of receiving monetary reward. Participants were allowed up to 4 s to make a decision. On observed trials, participants observed the ‘second player’s’ choices. At the beginning of each trial, one of two pairs of fractal images appeared on screen. Participants were told that in each trial, only one of the two presented cues would yield a reward, while the other will result in a neutral outcome. In the ‘Congruent’ pair, the outcome probabilities of the two cues were identical for both players (i.e., in both the experienced and the observed trials), and set to an 80:20 ratio (Fig. 1B). By contrast, the cues of the 'Incongruent' pair were associated with opposite choice-outcome probabilities (70:30 for the experienced trials and 30:70 for the observed trials). Participants were not informed about the possibility of similarities or differences in cue-outcome contingencies between the two pairs. We deliberately determined the probabilities associated with the congruent pair to be more easily learnt than those of the incongruent pair, to bias the participants into believing that the second participant was playing according to the same probabilities. To ensure that the actions and outcomes of the second player were sufficiently sampled, and at the same time to encourage reliability, we simulated the choices of the ‘second player’ such that they ‘chose’ the higher-outcome choice in 70% of the trials in the congruent pair and 50% in the incongruent pair. The second player’s choice appeared between 1550 and 2350 ms after trial onset. These reaction times were drawn pseudo-randomly from a list of 5 reaction times of a pilot test subject. To facilitate the initial stages of learning, the first 20 trials of the task consisted predominantly of experienced trials (18/20). After the first 20 trials, subjects were presented with 32 experienced trials and 48 observed trials, mixed in a random order. Rewards were drawn pseudo-randomly, i.e., the number of rewards for each choice type was pre-determined according to the contingencies, and their order randomized.

On each trial, after the choice was displayed on screen, a trial-unique picture, unrelated to the reward learning task, was presented at the center of the screen for 1.5 s (Fig. 1A). The pictures were taken from the neutral category of the International Affective Picture System (AIPS) database. To reveal the choice-related outcome, participants were required to press the ‘spacebar’ key. The presentation of a smiley emoji, superimposed on the picture, indicated the earning of 500 points, whereas a sad emoji indicated no reward. At the end of the experiment, the accumulated points were cashed in and paid as monetary reward.

### Recognition memory test

A surprise memory recognition test was administered immediately after the decision-making task (Fig. 1C). The test comprised presentation of the pictures that had been presented during the reward learning task, interleaved with new pictures that had never been presented. Participants were asked to indicate whether each presented picture had appeared before. Following each response, participants were required to indicate their degree of confidence regarding their previous answer, from 0—guess to 100—absolutely confident. The subjects were informed that performance on this phase would not be rewarded.

### Computational models

Participants' trial-by-trial choices were modeled using a RL algorithm^4^ and Softmax choice function^46^. Briefly, following each trial $t$, the value of the chosen stimulus $Q^{c}$ is updated according to the outcome of that trial:

$$ Q_{t + 1}^{c} = Q_{t}^{c} + \alpha *\delta_{t}^{c} $$

where $\alpha$ is the learning rate and $\delta^{c}$ is the reward prediction error, computed as the difference between predicted reward $Q^{c}$ and the actual reward $R$:

$$ \delta_{t} = R_{t} - Q_{t}^{c} $$

The value of the un-chosen stimulus $Q^{uc}$ was updated according to:

$$ Q_{t + 1}^{uc} = Q_{t}^{uc} + \alpha *\delta_{t}^{uc} $$

where $\delta^{uc}$ is computed as:

$$ \delta_{t}^{uc} = 1 - R_{t} - Q_{t}^{uc} $$

The values of all stimuli were set to an initial value of $0$. The reward $R$ was defined as $1$ for gains and as $0$ for neutral outcomes. The subjects’ choices on trial $t$ were modelled by the Softmax function, describing the probability of choosing stimulus $S$ from the variety of stimuli that were presented:

$$ p_{t} \left( {s = S} \right) = \frac{{e^{{\beta *Q_{t}^{s} }} }}{{\mathop \sum \nolimits_{i \in s} e^{{\beta *Q_{t}^{i} }} }} $$

where $\beta$ denotes the Softmax inverse temperature parameter, which controls the exploration element of each participant’s behavioral strategy. As $\beta$ becomes larger, choices are more deterministic.

For each participant, we fitted four learning models. In the single learning rate model, we estimated a single learning rate for experienced and observed trials, whereas in the two learning rates models we estimated separate learning rates either for experienced and observed trials, or for congruent and incongruent trials. In the fourth model, we estimated separate learning-rates for both experienced and observed trials as well as for congruent in incongruent conditions (four alpha model). The models’ parameters were optimized as those that maximized the likelihood of each subject’s choices using the Matlab built-in ‘fmin’ search function. We compared the four models for each subject using the Bayesian information criterion (BIC)^27^ for the entire population. Individual BIC scores were used to assign the subjects into one of the two strategies that best explained behavior—the ‘single strategy’ model, comprising subjects who were best fit by the single learning rate model, and the ‘divided strategy’ for subjects who were best fit by the two learning rates experienced/observed model. The remainder of the participants were labeled as unassigned, where the likelihood of the chosen model was not different than chance by at least one unit. In addition, we separated the subjects according to the relative difference in learning rates between experienced and observed trials. This analysis is presented in Supplementary Information and Figure S5.

To validate our model-choice approach, we computed a confusion matrix (Fig. S1), which included the four alternative models: a single learning-rate model, two two-learning-rate models (experienced/observed and congruent/incongruent), and a four learning-rate model. For each of the models we performed 100 simulations of groups of 60 subjects using randomly drawn parameters from the possible parameter range, and used our parameter estimation procedure, followed by model choice for each of these simulations.

The ability of the chosen model to accurately reflect the main effects of our subjects’ behavior was examined by posterior predictive checks (Fig. S2), in which we simulated behavioral choices of our subjects with their posterior distributions of the chosen model parameters reconstructing 95% confidence intervals of the learning curves of the different groups of subjects.

### Statistical analysis

Performance in the decision-making task was assessed by calculating the proportion of choices that each subject made of the cue associated with the highest reward outcome. Differences in these scores between congruent and incongruent conditions across subjects were assessed via a paired-sample t-test (across groups), and between subjects using a mixed model analysis, with strategy (single/divided) as a between-subject factor, and pair type (congruent/incongruent) as a within-subject factor. To explore the relationship between the decision-making scores and model fit quality, we carried out Pearson correlations between high-reward CS choice proportions and log likelihood of subject’s choices, separately for each group and each pair.

Memory performance was assessed by calculating for each subject the percentage of hits, misses, false alarms (FA), and correct rejections (CR) of the images presented during the surprise memory test. Overall memory strength was calculated by examining hits and FAs against chance level (i.e., 50%). To account for one’s tendency to provide positive answers, d prime (d′) values were calculated by subtracting normalized false-alarm rates from normalized hit rates for each participant. This measurement was used in a mixed-model repeated-measures analysis-of-variance (ANOVA) test, using subjects as a random effect, and trial type (experienced/observed), pair (congruent/incongruent), and group strategy (single/divided) as fixed factors. Interactions between memory performance and model fit were examined by performing Pearson correlations between log-likelihood (as a measure of model fit quality) and corrected hit-rates.

To validate the use of highest available cue value at each trial as a possible predictor of memory performance (see below), we computed correlations between 20 bins of high-reward values and mean choice reaction times (RT) for those bins. Correlation values were transformed into normal distributions by using a Fisher z-transformation. To enable cross-subject analysis, the computed cue-values were normalized for each subject using a standard z-score calculation. For all correlation analysis, bins with less than 20 cases were omitted from the correlation computation. To test for significance of the relationship between the reported variables, we used a permutation approach, whereby for each subject, the mean RT values of cue-value bins were randomly shuffled 10,000 times and correlated with the cue-value. This resulted in the distribution of correlations under the null hypothesis. This distribution served to compute the *p* value, defined as the proportion of correlations above/below the actual correlation coefficient between the variables. A similar permutation approach was applied for exploring the relationship between memory performance (proportion of hit responses), as well as for confidence ratings, and computed cue values, separately for each model group and for each condition (experienced/observed trials).

To compare correlation coefficients between the 'single' and 'divided' groups we used Fisher Z transformation. To test differences between correlation coefficients of the same subjects in experienced and observed trials we applied Meng's Z test for dependent correlations^30^.

## Supplementary Information

Supplementary Information.
