# Supplementary material for: Individual differences in experienced and observational decision-making illuminate interactions between reinforcement learning and declarative memory
Source: Sci Rep. 2021 Mar 15;11:5899. doi: 10.1038/s41598-021-85322-2 (PMC7971018; doi:10.1038/s41598-021-85322-2)
Supplement: Supplementary file 1 — Supplementary Information. [file 41598_2021_85322_MOESM1_ESM.docx]

**Supplementary Information**

**Individual differences in experienced and observational decision-making illuminate interactions between reinforcement learning and declarative memory**

Batel Yifrah, Ayelet Harmaty, Genela Morris & Avi Mendelsohn

**
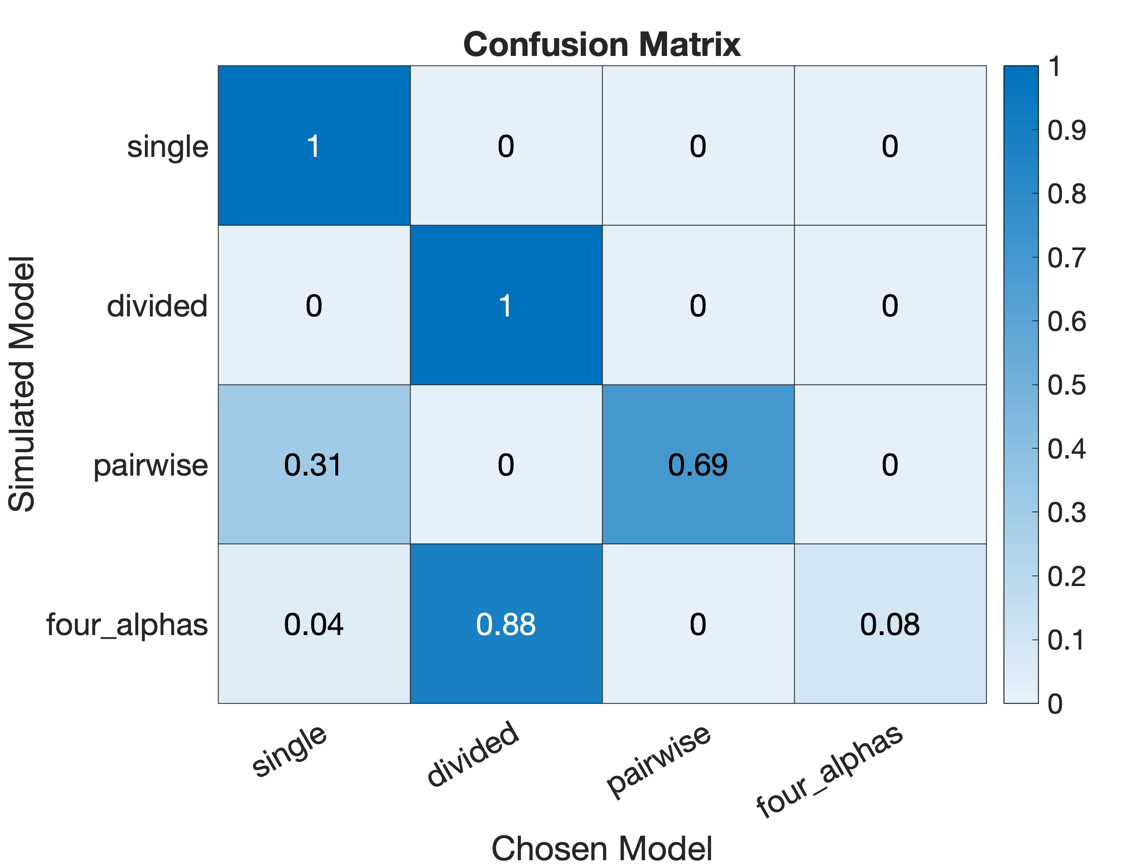
**

**Figure S1. Confusion matrix.** Each row of the matrix depicts the frequencies with which each of the alternative models is chosen for simulated behavior generated under a given model. The matrix diagonal represents model recovery, whereas off-diagonal cells reflect model confusion. The matrix demonstrates that our model choice procedure efficiently captured the single, divided, and pairwise (congruent/incongruent) models. The four-learning-rate model, in contrast, was not recovered, and was instead explained by the two-learning-rate ‘divided’ model, indicating that there was not sufficient power to maintain this model. Note that the confusion matrix demonstrates the bias in favoring models with fewer parameters.

**
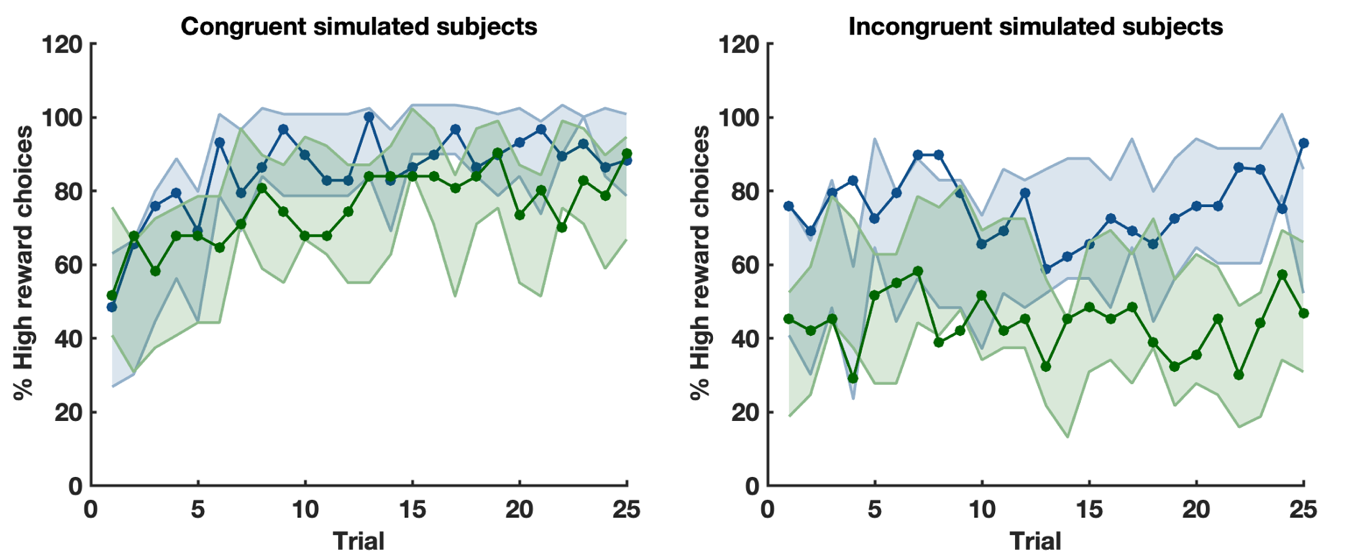
**

**Figure S2.** **Posterior predicitve checks.** Behavioral data for the ‘single’ (green) and ‘divided’ (blue) strategy groups are shown in solid lines alongside the 95 % confidence interval of simulated data that was generated using the posterior distributions of model parameters. This analysis is shown separately for congruent (right panel) and incongruent (left panel) conditions.


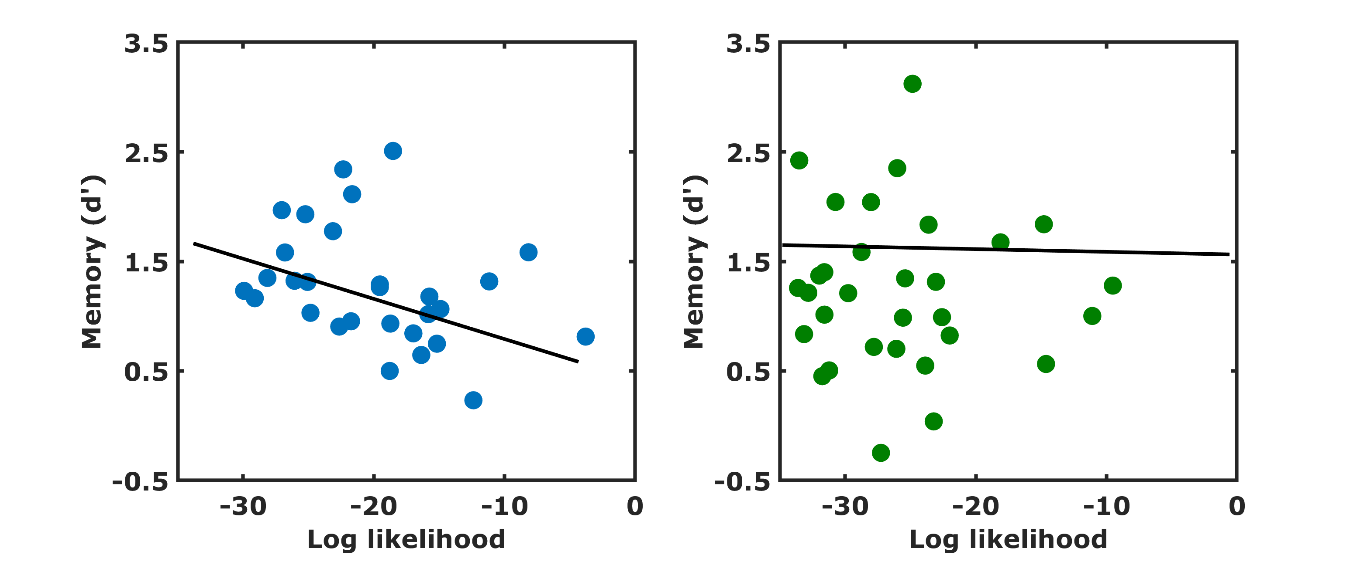


**Figure S3. Reinforcement learning model fits and memory performance in the ‘single strategy’ and ‘divided strategy’ groups.** (**A**) In the ‘divided strategy’ group, reinforcement learning model fit (log likelihood) showed a trend towards negative correlation with declarative memory (d’). (**B**) No correlation was found in the ‘single strategy’ group.


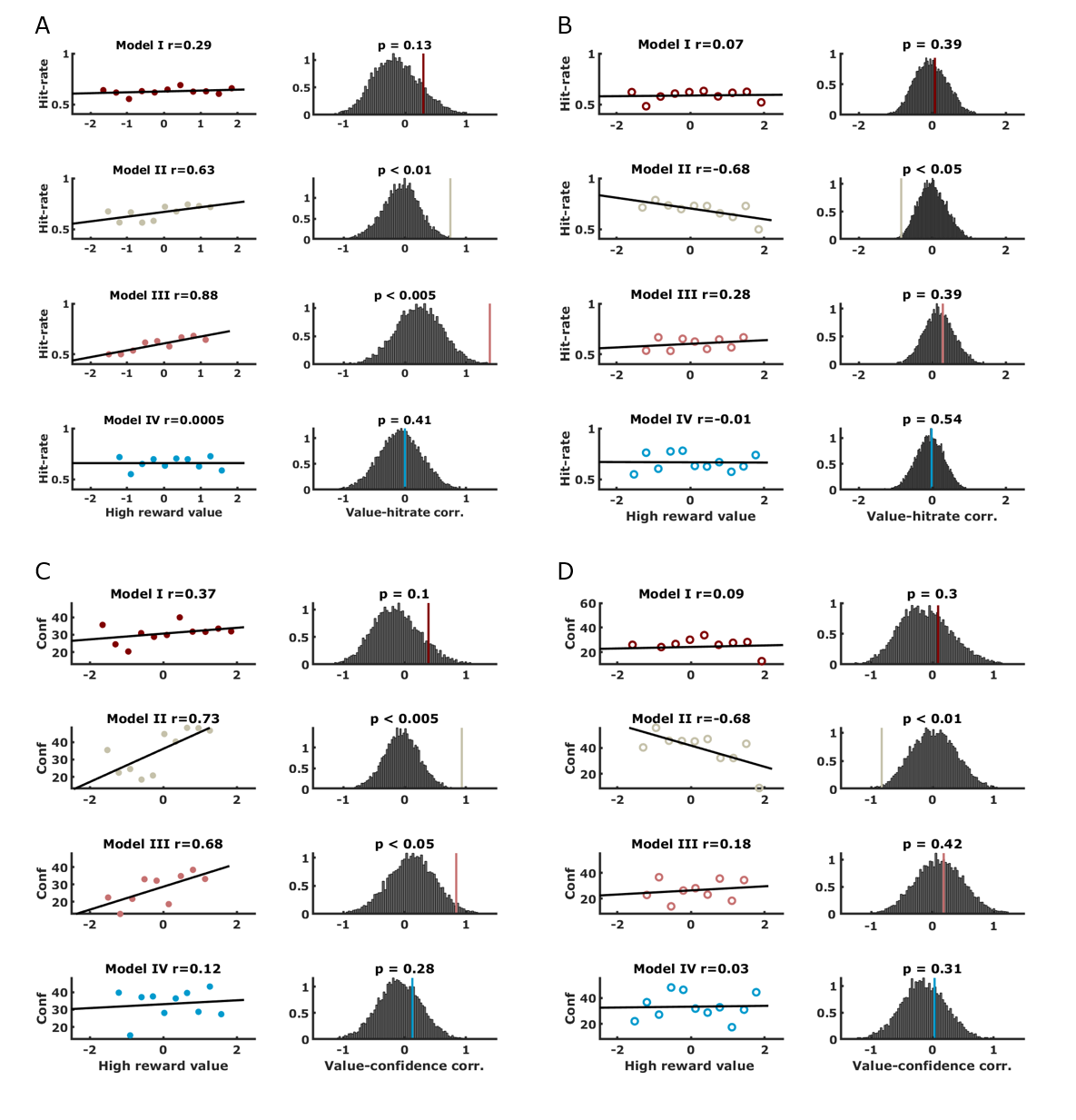


**Figure S4. Memory and confidence analysis according to hypothetical interpretations of observed stimulus-feedback contingencies** *Memory performance and confidence ratings are associated with maximum cue-values during experienced trials of participants with large differential learning rates.* (**A, B**) Scatter plots depicting correlations between memory performance and the maximum stimulus-value available in a trial, similar to the analysis presented in Figs. 4 & 5 in the main text). Participants are divided into the best fitting model out of four alternatives of stimulus-feedback contingencies of observed trials, as described in Table S2. Data is shown for experienced trials (left panels) and observed trials (right panels). (**C, D**) Similar analysis as for (A) and (B) for correlations between maximum stimulus-values with confidence ratings for correspnding images. See Table S1,2 for details of model assignment


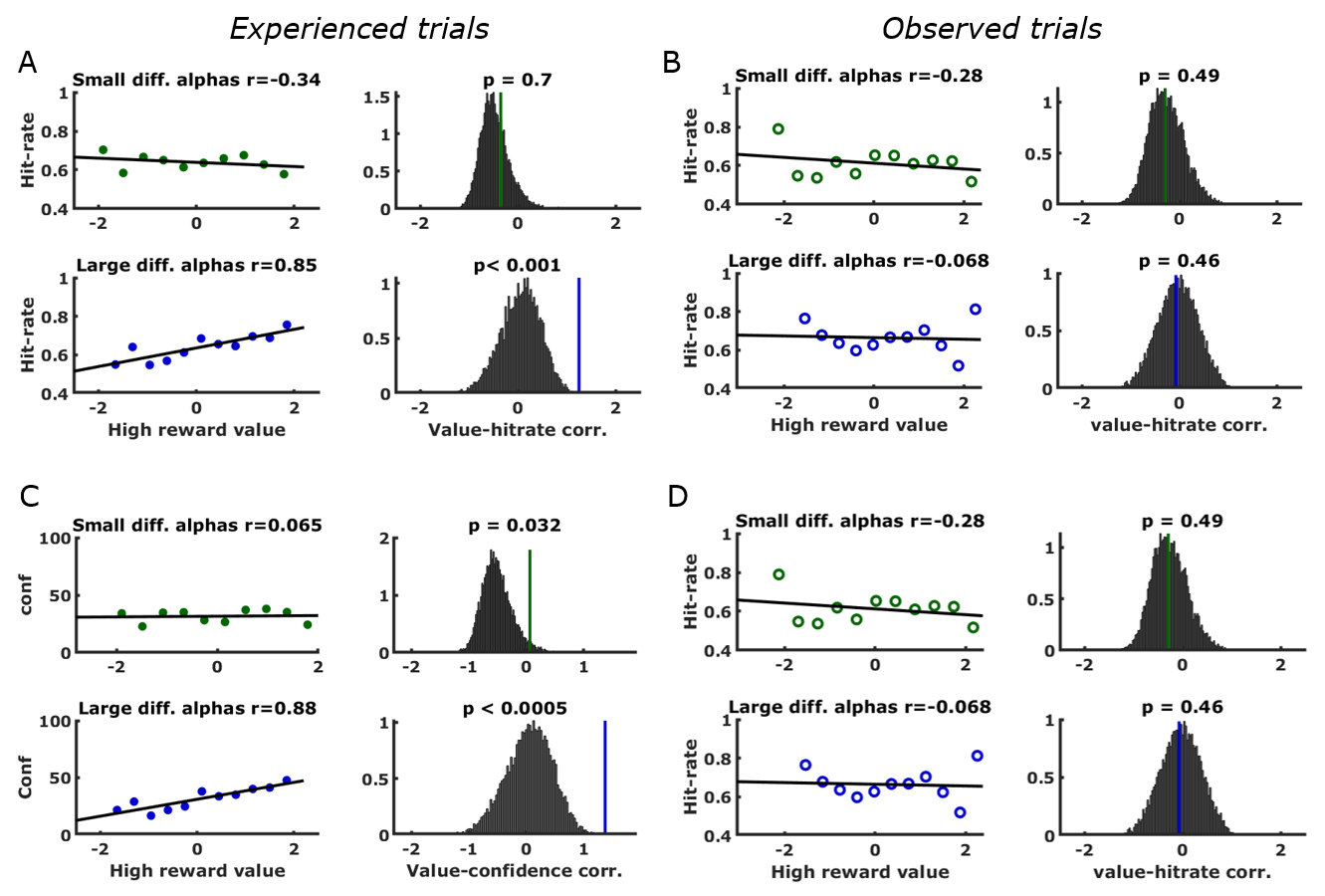


**Figure S5. Memory and confidence analysis according to relative learning rate division of subjects** *Memory performance and confidence ratings are associated with maximum cue-values during experienced trials of participants with large differential learning rates.* (**A, B**) Scatter plots depicting correlations between memory performance and the maximum stimulus-value available in a trial (see also Figs. 4 & 5 in the main text). Participants are divided into those with small differences in learning rates for experienced vs. observed conditions (<three-fold difference, green scatter plots) and those with large differential learning rates (> three-fold difference, blue scatter plots). Data is shown for experienced trials (left panels) and observed trials (right panels). (**C, D**) Similar analysis as for (A) and (B) for correlations between maximum stimulus-values with confidence ratings for correspnding images.

**Table S1. Model parameters**

| Sub | $\boldsymbol{\alpha(exp)}$ | $\boldsymbol{\alpha(obs)}$ | $\boldsymbol{\beta}$ | LogL | BIC 1$\boldsymbol{\alpha}$ | BIC  2$\boldsymbol{\alpha}$  Exp/Obs | BIC  $\mathbf{2}\boldsymbol{\alpha}$  Con/Incon | BIC 4$\boldsymbol{\alpha}$ | Chosen model | Chosen pair model | BIC pair model |
| --- | --- | --- | --- | --- | --- | --- | --- | --- | --- | --- | --- |
| 1 | 0.1109 | 0.0683 | 4.56 | -23.32 | 58.31 | 55.53 | 56.83 | 64.26 | Single | Model1 | 58.31 |
| 2 | 0.1717 | 0.4088 | 3.72 | -23.47 | 58.67 | 53.01 | 60.32 | 65.38 | Single | Model1 | 58.67 |
| 3 | 0.8696 | 0.3005 | 1.39 | -24.43 | 60.06 | 59.50 | 63.24 | 67.50 | Single | Model3 | 58.58 |
| 4 | 0.0389 | 0.0547 | 20.91 | -9.45 | 30.26 | 26.61 | 28.75 | 35.88 | Single | Model1 | 30.26 |
| 5 | 0.0011 | 0.0000 | 99.93 | -30.51 | 72.56 | 70.89 | 71.87 | 80.91 | Single | Model2 | 69.37 |
| 6 | 0.5309 | 0.5886 | 1.63 | -26.08 | 63.64 | 59.82 | 58.04 | 64.46 | Single | Model1 | 63.64 |
| 7 | 0.0784 | 0.0322 | 16.44 | -9.59 | 30.92 | 30.01 | 31.70 | 37.90 | Single | Model1 | 30.92 |
| 8 | 0.0028 | 0.0014 | 99.92 | -23.81 | 59.30 | 58.94 | 60.89 | 65.32 | Single | Model1 | 59.30 |
| 9 | 0.0938 | 1.0000 | 0.62 | -33.10 | 77.94 | 75.03 | 78.91 | 83.45 | Single | Model1 | 77.94 |
| 10 | 0.1549 | 0.0145 | 2.29 | -29.80 | 71.28 | 71.27 | 73.90 | 78.56 | Single | Model3 | 69.95 |
| 11 | 0.1856 | 0.1252 | 4.93 | -21.61 | 54.90 | 51.79 | 54.42 | 56.57 | Single | Model3 | 47.31 |
| 12 | 0.0017 | 0.0007 | 99.99 | -27.54 | 66.77 | 65.28 | 67.44 | 74.45 | Single | Model1 | 66.77 |
| 13 | 0.0136 | 0.1144 | 5.16 | -22.43 | 56.59 | 55.06 | 52.28 | 65.79 | Single | Model1 | 56.59 |
| 14 | 0.3141 | 0.7708 | 2.22 | -23.64 | 58.90 | 58.57 | 62.27 | 65.99 | Single | Model1 | 58.90 |
| 15 | 0.0038 | 0.0021 | 100.0 | -23.11 | 57.96 | 57.51 | 57.17 | 64.86 | Single | Model1 | 57.96 |
| 16 | 0.8692 | 0.9005 | 1.19 | -28.02 | 67.71 | 63.82 | 66.07 | 71.50 | Single | Model1 | 67.71 |
| 17 | 0.4922 | 0.8478 | 0.74 | -32.93 | 77.61 | 74.11 | 78.01 | 83.83 | Single | Model1 | 77.61 |
| 18 | 0.9873 | 0.6347 | 0.53 | -33.41 | 78.56 | 74.84 | 78.13 | 84.60 | Single | Model3 | 76.80 |
| 19 | 0.3123 | 0.5692 | 0.77 | -31.43 | 74.42 | 70.87 | 70.63 | 79.48 | Single | Model4 | 74.29 |
| 20 | 0.1725 | 0.1140 | 2.91 | -27.09 | 65.91 | 62.34 | 60.67 | 66.22 | Single | Model1 | 65.91 |
| 21 | 0.7573 | 0.1615 | 0.74 | -32.49 | 76.65 | 73.43 | 76.99 | 84.04 | Single | Model2 | 76.07 |
| 22 | 0.0029 | 0.0019 | 98.77 | -22.30 | 56.28 | 54.23 | 49.92 | 57.27 | Single | Model1 | 56.28 |
| 23 | 1.0000 | 0.8506 | 0.49 | -30.73 | 72.94 | 69.13 | 72.96 | 78.21 | Single | Model3 | 68.66 |
| 24 | 1.0000 | 0.2301 | 0.88 | -29.67 | 70.88 | 70.14 | 71.11 | 77.25 | Single | Model1 | 70.88 |
| 25 | 0.2928 | 0.1669 | 2.43 | -27.32 | 66.20 | 63.31 | 66.74 | 73.58 | Single | Model1 | 66.20 |
| 26 | 0.0870 | 0.1437 | 7.83 | -14.34 | 40.42 | 37.09 | 40.65 | 46.80 | Single | Model1 | 40.42 |
| 27 | 0.0958 | 0.3577 | 1.97 | -28.94 | 69.61 | 67.35 | 70.26 | 75.01 | Single | Model1 | 69.61 |
| 28 | 0.0379 | 0.0210 | 23.71 | -13.00 | 37.54 | 37.24 | 39.68 | 43.82 | Single | Model1 | 37.54 |
| 29 | 0.1658 | 0.0900 | 5.60 | -17.61 | 46.89 | 44.01 | 45.97 | 52.67 | Single | Model1 | 46.89 |
| 30 | 0.3720 | 0.6457 | 2.51 | -22.13 | 55.87 | 53.86 | 57.62 | 63.01 | Single | Model1 | 55.87 |
| 31 | 0.7704 | 0.7678 | 0.70 | -31.98 | 63.73 | 66.47 | 75.11 | 71.46 | Single | Model2 | 75.07 |
| 32 | 0.0021 | 0.0000 | 99.94 | -26.06 | 71.52 | 71.77 | 75.25 | 78.97 | Divided | Model4 | 63.61 |
| 33 | 0.6376 | 0.0000 | 1.13 | -29.92 | 55.05 | 66.81 | 71.84 | 62.26 | Divided | Model4 | 71.52 |
| 34 | 0.1731 | 0.0000 | 3.97 | -21.66 | 65.82 | 68.45 | 66.13 | 64.97 | Divided | Model4 | 54.66 |
| 35 | 0.0650 | 0.0036 | 6.69 | -27.04 | 61.88 | 70.11 | 69.14 | 68.63 | Divided | Model2 | 60.60 |
| 36 | 0.0028 | 0.0000 | 99.98 | -25.07 | 62.09 | 65.99 | 69.64 | 67.71 | Divided | Model2 | 60.19 |
| 37 | 0.1053 | 0.0000 | 3.63 | -25.24 | 50.56 | 65.82 | 60.55 | 54.65 | Divided | Model2 | 61.51 |
| 38 | 0.0035 | 0.0000 | 99.99 | -19.54 | 68.01 | 74.11 | 77.90 | 75.79 | Divided | Model2 | 46.14 |
| 39 | 0.2057 | 0.0000 | 2.52 | -28.14 | 41.54 | 66.76 | 64.70 | 30.98 | Divided | Model4 | 57.62 |
| 40 | 0.0493 | 0.0000 | 13.25 | -14.90 | 45.62 | 53.85 | 54.99 | 52.79 | Divided | Model2 | 35.11 |
| 41 | 0.0432 | 0.0092 | 14.45 | -16.97 | 65.26 | 68.50 | 65.88 | 71.32 | Divided | Model1 | 45.62 |
| 42 | 0.0356 | 0.0023 | 10.02 | -26.79 | 19.14 | 58.95 | 48.34 | 26.70 | Divided | Model2 | 65.07 |
| 43 | 0.0380 | 0.0070 | 100 | -3.79 | 42.98 | 61.98 | 56.25 | 48.89 | Divided | Model1 | 19.14 |
| 44 | 0.1432 | 0.0121 | 8.45 | -15.75 | 48.67 | 56.56 | 55.54 | 56.24 | Divided | Model3 | 41.48 |
| 45 | 0.1135 | 0.0132 | 8.19 | -18.53 | 49.05 | 54.92 | 56.82 | 52.84 | Divided | Model1 | 48.67 |
| 46 | 0.1897 | 0.0239 | 4.25 | -18.78 | 44.35 | 47.82 | 49.77 | 48.25 | Divided | Model3 | 48.28 |
| 47 | 0.0000 | 0.1399 | 7.83 | -16.37 | 36.37 | 52.86 | 56.67 | 40.38 | Divided | Model1 | 44.35 |
| 48 | 0.1150 | 0.0098 | 10.95 | -12.38 | 55.22 | 61.14 | 65.05 | 62.08 | Divided | Model1 | 36.37 |
| 49 | 0.0075 | 0.0027 | 99.78 | -21.74 | 49.14 | 54.00 | 53.66 | 52.26 | Divided | Model3 | 53.35 |
| 50 | 0.0056 | 0.0024 | 99.87 | -18.73 | 34.01 | 41.04 | 28.76 | 32.14 | Divided | Model1 | 49.14 |
| 51 | 0.3169 | 0.1273 | 8.72 | -11.17 | 61.36 | 64.54 | 64.80 | 67.95 | Divided | Model1 | 34.01 |
| 52 | 0.6749 | 0.0450 | 1.84 | -24.84 | 42.08 | 45.45 | 48.96 | 49.59 | Divided | Model2 | 61.26 |
| 53 | 0.1765 | 0.0599 | 9.46 | -15.17 | 57.98 | 62.62 | 54.61 | 59.82 | Divided | Model1 | 42.08 |
| 54 | 0.0028 | 0.0008 | 99.97 | -23.12 | 50.75 | 58.48 | 55.83 | 52.94 | Divided | Model2 | 57.04 |
| 55 | 0.2012 | 0.0386 | 6.17 | -19.54 | 56.35 | 64.30 | 67.76 | 60.07 | Divided | Model1 | 50.75 |
| 56 | 0.3374 | 0.0000 | 3.63 | -22.34 | 56.96 | 63.92 | 66.67 | 63.01 | Divided | Model2 | 55.28 |
| 57 | 0.0682 | 0.0000 | 5.81 | -22.64 | 43.27 | 55.64 | 44.88 | 46.61 | Divided | Model4 | 55.09 |
| 58 | 0.0397 | 0.0089 | 17.16 | -15.83 | 69.88 | 73.04 | 75.91 | 76.62 | Divided | Model1 | 43.27 |
| 59 | 1.0000 | 0.0000 | 0.96 | -29.10 | 28.00 | 48.45 | 51.63 | 28.53 | Divided | Model4 | 68.73 |
| 60 | 0.0115 | 0.0026 | 100 | -8.16 | 63.73 | 66.47 | 66.42 | 71.46 | Divided | Model1 | 28.00 |

The Bic1α, Bic2α, Bic4α, and chosen model columns refer to parameters of the models allowing for either one, two or four learning rates. There are two two-learning-rate models, one referring to separate learning rates for experienced and observed trials, and the other for congruent and incongruent trials. The Chosen pair model and BIC pair models refer to the best fitting model out of four alternative models according to different hypothetical interpretations of stimulus-outcome contingencies in observed trials (see also Table S2). BIC – Bayesian information criterion, Exp – Experienced, Obs – Observed, Con – Congruent, Incon – Incongruent.

**Table S2. Description of alternative modeling approach, allowing for two learning-rates that account for various hypothetical interpretations of observed stimulus-feedback contingencies.**

| **Model** | **Congruent pair** | **Incongruent pair** |
| --- | --- | --- |
| I | **Same** contingency for subject and 2^nd^ player | **Same** contingency for subject and 2^nd^ player |
| II | **Same** contingency for subject and 2^nd^ player | **Reversed** contingency for subject and 2^nd^ player |
| III | **Reversed** contingency for subject and 2^nd^ player | **Same** contingency for subject and 2^nd^ player |
| IV | **Reversed** contingency for subject and 2^nd^ player | **Reversed** contingency for subject and 2^nd^ player |

**Table S3. Log likelihoods for ‘unassigned’ participants.**

| Subject | Log likelihood – single strategy model | Log likelihood – divided strategy model | Chosen pair model |
| --- | --- | --- | --- |
| 1 | -33.27 | -33.27 | Model3 |
| 2 | -32.58 | -32.30 | Model2 |
| 3 | -32.57 | -31.67 | Model4 |
| 4 | -34.16 | -32.85 | Model2 |
| 5 | -33.96 | -33.90 | Model4 |
| 6 | -30.22 | -30.14 | Model2 |
| 7 | -34.45 | -34.30 | Model2 |
| 8 | -33.90 | -33.90 | Model3 |
| 9 | -31.70 | -31.15 | Model3 |
| 10 | -32.75 | -31.91 | Model4 |
| 11 | -24.95 | -24.33 | Model4 |
| 12 | -33.27 | -32.21 | Model4 |
| 13 | -31.69 | -31.41 | Model1 |
| 14 | -33.27 | -33.27 | Model4 |
| 15 | -34.49 | -34.18 | Model4 |

See Table S2 for description of the models indicated in the chosen pair model column.

**Instructions to participants (translated from Hebrew)**

Hello and thank you for your participation.

In the following experiment, two people will participate - yourself and another participant.

During the experiment, you and the additional participant will be able to gain monetary rewards, which will be added to your payment for participation in this experiment.

**Any monetary gain gained during the experiment will be divided equally between you and the additional participant.**

The experiment consists of two sequential stages.

The first stage of the experiment consists of 100 steps and will last about twenty minutes.

During this stage, at each step, you will be presented with one of two pairs of figures. In each step, you will be asked to choose one of the figures by pressing on a corresponding keyboard button. Each figure in the pair has a predetermined probability for winning, and the second figure has the complementary one.

For example, if in the first pair figure A leads to winning in 60% of the steps, figure B will lead to winning in 40% of the steps.

At each step, choosing one of the figures will yield 500 points, while choosing the second figure will give a feedback of 0 points. Your task is to accumulate as many points as possible.

In some of the steps you will be asked to choose between the pairs of figures while in the remaining rounds, the other player will be asked to decide and his choices will be displayed on screen.

If within 4 seconds no figure is selected, the words "Time's up" will appear on the screen and the experiment will continue by pressing the space bar.

**In order to optimize your choices and thereby increase your profits, try learning the winning probability of each figure while playing**.

After selecting one of the figures, an image will appear on the screen while waiting for the choice feedback. To see if you won or not, you will be asked to press the space bar.

The images do not affect your chances of winning and their role is solely to pass the time while waiting.

Throughout the experiment, the total amount of points you had accumulated up to that stage will appear at the bottom of the screen.

At the end of the experiment, each 500 points you had earned will be converted to 3 NIS, which you will receive at the end of the experiment in addition to the amount that was set in advance for participating in the experiment. The maximum amount that can be earned for the experiment is 80 NIS.

Between rounds, a white plus will appear in the center of the screen. Please focus your gaze on it at the time of its appearance.

Once this stage is complete, the second stage of the experiment will begin with instructions and explanations.

Good luck!
